# Effectiveness of SGLT2is vs. GLP-1RAs on cardiovascular and cerebrovascular outcomes in T2D patients according to CVD status

**DOI:** 10.3389/fcvm.2022.1011535

**Published:** 2022-09-06

**Authors:** Lixin Du, Zhigang Li, Peng Lan, Huayu Huang, Wende Cheng

**Affiliations:** ^1^Department of Medical Imaging, Shenzhen Longhua District Central Hospital, Shenzhen, China; ^2^Department of Pathology, Shenzhen Longhua District Central Hospital, Shenzhen, China

**Keywords:** SGLT2is, GLP-1RAs, type 2 diabetes, cardiovascular, stroke, death

## Introduction

In a population-based cohort study (1), Dong et al. assessed the comparative cardiovascular effectiveness of sodium-glucose cotransporter 2 inhibitors (SGLT2is) vs. glucagon-like peptide-1 receptor agonists (GLP-1RAs) in patients with type 2 diabetes (T2D) according to baseline status of cardiovascular disease (CVD) and chronic kidney disease (CKD). The authors concluded that SGLT2is and GLP-1RAs seemed to have comparable effectiveness on myocardial infarction (MI) and total stroke overall but their comparative effectiveness might vary in different patient subgroups. Due to the limited statistical power, the authors state in their Conclusion section that those findings from subgroup analyses need to be further investigated. Hence, we intended to conduct a further meta-analysis to validate and extend those findings deriving from the subgroup analyses according to baseline status of CVD in Dong et al.' article (1).

## Methods

This meta-analysis was performed on the basis of the statement of Preferred Reporting Items for Systematic Reviews and Meta-Analyses (PRISMA) (2). We searched Web of science, Embase, and PubMed from inception date to July 2022. The main search keywords were: “type 2 diabetes”, “SGLT2 inhibitors”, “GLP-1RAs”, “cardiovascular”, “stroke”, “death”, “real-world”, and “cohort study”. Studies eligible to inclusion were cohort studies that assessed the relative effectiveness of SGLT2is vs. GLP-1RAs on cardiovascular or cerebrovascular outcomes in T2D patients. We excluded those studies in which the subgroup analyses according to baseline CVD status were not performed on the outcomes of interest. The outcomes of interest consisted of major adverse cardiovascular events (MACE), heart failure (HF), cardiovascular mortality (CVM), MI, stroke, and all-cause mortality (ACM). MACE was defined as a composite of non-fatal MI, non-fatal stroke, and CVM; or a composite of fatal and non-fatal MI, and fatal and non-fatal stroke; but not a composite of MI, stroke, and ACM. We did random-effects meta-analyses using the adjusted hazard ratios (HRs) and 95% confidence intervals (CIs) extracted from included studies. When HRs and their 95% CIs were not available from original studies, we used risk ratios (RRs) and their 95% CIs instead. Subgroup analyses were done in accordance with baseline CVD status. CVD was defined as cerebrovascular disease, coronary heart disease, peripheral vascular disease, or HF (3). We assessed subgroup differences by Cochran's Q test, and evaluated publication bias by Egger test and funnel plots. *P* < 0.05 was considered as statistical significance. Data analyses were conducted using Stata/MP (Version 16.0).

## Results

**Figure 1:**
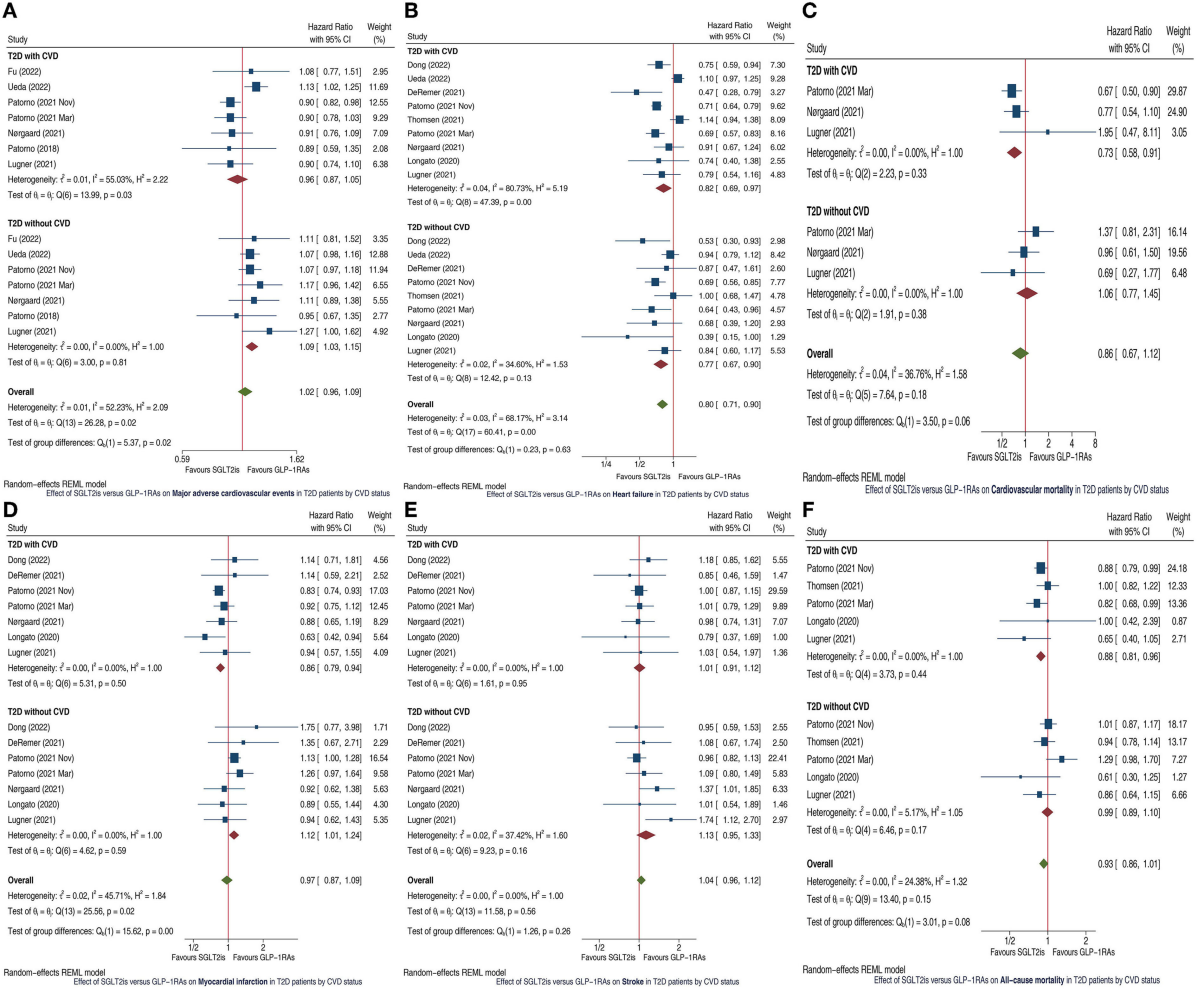
Forest plots showing the effects of SGLT2is vs. GLP-1RAs on major adverse cardiovascular events **(A)**, heart failure **(B)**, cardiovascular mortality **(C)**, myocardial infarction **(D)**, stroke **(E)**, and all-cause mortality **(F)** in T2D patients by CVD status. SGLT2is, sodium-glucose cotransporter 2 inhibitors; GLP-1RAs, glucagon-like peptide-1 receptor agonists; T2D, type 2 diabetes; CVD, cardiovascular disease; CI, confidence interval.

After preliminary screening we identified 18 cohort studies (1, 3–19) which compared cardiovascular outcomes between GLP-1RAs and SGLT2is in patients with T2D. After reading the full articles of these 18 studies, we finally included 11 studies (1, 3–12) in this meta-analysis, whereas we excluded the other 7 studies (13–19) since they did not report relevant subgroup analyses for the outcomes of our interest. SGLT2is vs. GLP-1RAs had similar MACE risk in T2D patients with CVD (HR 0.96, 95% CI 0.87-1.05), but was significantly associated with higher MACE risk in those without CVD (HR 1.09, 95% CI 1.03–1.15); and the subgroup difference was statistically significant (*P* =0.02; Figure 1A). SGLT2is vs. GLP-1RAs was significantly associated with lower HF risk in T2D patients with CVD (HR 0.82, 95% CI 0.69-0.97) and without CVD (HR 0.77, 95% CI 0.67–0.90), and the subgroup difference had no statistical significance (*P* =0.63; Figure 1B). SGLT2is vs. GLP-1RAs was significantly associated with lower CVM risk in T2D patients with CVD (HR 0.73, 95% CI 0.58–0.91), but had similar CVM risk in those without CVD (HR 1.06, 95% CI 0.77–1.45); and the subgroup difference approximated to statistical significance (*P* =0.06; Figure 1C). SGLT2is vs. GLP-1RAs was significantly associated with lower MI risk in T2D patients with CVD (HR 0.86, 95% CI 0.79–0.94), but with higher MI risk in those without CVD (HR 1.12, 95% CI 1.01–1.24); and the subgroup difference was statistically significant (*P* < 0.01; Figure 1D). SGLT2is vs. GLP-1RAs had similar stroke risk in T2D patients with CVD (HR 1.01, 95% CI 0.91–1.12) and without CVD (HR 1.13, 95% CI 0.95–1.33), and the subgroup difference had no statistical significance (*P* = 0.26; Figure 1E). SGLT2is vs. GLP-1RAs was significantly associated with lower ACM risk in T2D patients with CVD (HR 0.88, 95% CI 0.81–0.96), but had similar ACM risk in those without CVD (HR 0.99, 95% CI 0.89–1.10); and the subgroup difference approximated to statistical significance (*P* = 0.08; Figure 1F). The funnel plots and Egger test results (Supplementary Figure S1) suggested no obvious publication bias for all the outcomes of interest (*P*_*Egger*_ ranged from 0.208 to 0.900).

## Discussion

This meta-analysis yielded three main findings. First, our meta-analysis confirmed the findings regarding to HF and stroke in Dong et al.' study (1): SGLT2is vs. GLP-1RAs had lower HF risk and similar risk of total stroke in T2D patients regardless of CVD status. Second, our meta-analysis updated the finding regarding to MI in Dong et al.' study (1). Dong et al. found that the two drug classes had similar MI risk irrespective of CVD status, whereas we found that SGLT2is vs. GLP-1RAs had lower MI risk in T2D patients with CVD but had higher MI risk in those without CVD. This updated finding might be attributed to the superiority of our meta-analysis over Dong et al.' study (1) in statistical power. Last, our meta-analysis extended the findings of Dong et al.' study (1) by assessing three new outcomes: MACE, CVM, and ACM. Dong et al. failed to assess these three outcomes, whereas we assessed these and identified the following new findings: SGLT2is vs. GLP-1RAs had lower risks of CVM and ACM in T2D patients with CVD but had similar risks in those without CVD; and SGLT2is vs. GLP-1RAs had similar MACE risk in T2D patients with CVD but had higher MACE risk in those without CVD.

To our knowledge, this is the first meta-analysis which evaluated the comparative effectiveness of SGLT2is vs. GLP-1RAs on several important cardiovascular/cerebrovascular outcomes in T2D patients according to baseline CVD status. Its findings suggest that SGLT2is might be preferred to GLP-1RAs in reducing CVM, ACM and MI in T2D patients with CVD; whereas GLP-1RAs might be preferred to SGLT2is in reducing MACE and MI in those without CVD. After being confirmed by future randomized trials, these findings would help to select between SGLT2is and GLP-1RAs in specific clinical settings. On the contrary, we only compared risk of total stroke between SGLT2is and GLP-1RAs, but failed to compare risks of stroke subtypes, i.e., ischemic stroke and hemorrhagic stroke, due to the limited data. Further studies are needed to address this issue. Moreover, the mechanisms by which the relative effectiveness of SGLT2is vs. GLP-1RAs varies in T2D patients with/without CVD are required to be further investigated.

In conclusion, our meta-analysis validated the findings regarding to HF and stroke in Dong et al.' study (1). Moreover, we additionally revealed the potential superiority of SGLT2is over GLP-1RAs in reducing CVM, ACM and MI in T2D patients with CVD and the potential superiority of GLP-1RAs over SGLT2is in reducing MACE and MI in those without CVD. After being confirmed by future randomized trials, these findings would help to select between the two drug classes in specific clinical settings.

## Summary

In a population-based cohort study (1), Dong et al. assessed the comparative cardiovascular effectiveness of sodium-glucose cotransporter 2 inhibitors (SGLT2is) vs. glucagon-like peptide-1 receptor agonists (GLP-1RAs) in patients with type 2 diabetes (T2D) according to baseline status of cardio-renal disease. The authors concluded that those findings from subgroup analyses in their article needed to be further investigated, due to the limited statistical power. In order to confirm and extend Dong et al.'s findings, we conducted a further meta-analysis by incorporating Dong et al. 's study and previous relevant studies. Our meta-analysis validated the findings regarding to heart failure and stroke in Dong et al.' study (1). More importantly, we additionally revealed the potential superiority of SGLT2is over GLP-1RAs in reducing death and myocardial infarction (MI) in T2D patients with cardiovascular disease (CVD) and the potential superiority of GLP-1RAs over SGLT2is in reducing major adverse cardiovascular events and MI in those without CVD. After being confirmed by future randomized trials, these findings would help to select between the two drug classes in specific clinical settings. This is a commentary on a previous article published outside of Frontiers. Therefore, we submitted this manuscript as an Opinion article, as suggested in the Author Guidelines.

## Author contributions

Design: LD. Conduct/data collection: PL, HH, and WC. Analysis: PL. Writing manuscript: LD and ZL. All authors approved the manuscript.

## Funding

This work was supported by the Key Laboratory of Neuroimaging, Longhua District, Shenzhen [Shen Long Hua Ke Chuang Ke Ji Zi (2022) No. 7] and Shenzhen Fundamental Research Program (Natural Science Foundations), General Program for Fundamental Research (Grant No. JCYJ20210324142404012).

## Conflict of interest

The authors declare that the research was conducted in the absence of any commercial or financial relationships that could be construed as a potential conflict of interest.

## Publisher's note

All claims expressed in this article are solely those of the authors and do not necessarily represent those of their affiliated organizations, or those of the publisher, the editors and the reviewers. Any product that may be evaluated in this article, or claim that may be made by its manufacturer, is not guaranteed or endorsed by the publisher.

## Supplementary material

Supplementary Figure S1 Funnel plots including Egger test results for 6 outcomes of interest.

## Abbreviations

SGLT2is: sodium-glucose cotransporter 2 inhibitors
GLP-1RAs: glucagon-like peptide-1 receptor agonists
T2D: type 2 diabetes
CVD: cardiovascular disease
CKD: chronic kidney disease
MI: myocardial infarction
MACE: major adverse cardiovascular events
HF: heart failure
CVM: cardiovascular mortality
ACM: all-cause mortality
HR: hazard ratio
CI: confidence interval
RR: risk ratio.
